# A TLR4-Associated Microglia–Endothelial Inflammatory Axis in Drug-Resistant Temporal Lobe Epilepsy: Integrated Transcriptomic and Genetic Evidence

**DOI:** 10.3390/ijms27219960

**Published:** 2026-11-06

**Authors:** Zhenglin Liu, Jian Yin

**Affiliations:** The Second Affiliated Hospital of Dalian Medical University, Dalian 116027, China; liuzl02@dmu.edu.cn

**Keywords:** drug-resistant temporal lobe epilepsy, neuroinflammation, toll-like receptor 4, microglia, single-cell/nucleus RNA sequencing, cell–cell communication, blood–brain barrier, kynurenine, diagnostic biomarker, drug repurposing

## Abstract

Microglia-driven neuroinflammation contributes to epileptogenesis and drug resistance in temporal lobe epilepsy (TLE), but the cellular organization of the neuroinflammatory hub and its coupling to the cerebrovascular endothelium remain unresolved. We performed a retrospective secondary analysis of publicly available datasets, integrating paired hippocampal bulk transcriptomics (GSE134697), an integrated temporal-lobe single-cell/nucleus RNA sequencing (sc/snRNA-seq) atlas (9 patients with drug-resistant TLE and 4 controls), two-sample Mendelian randomization (MR) and summary-based Mendelian randomization (SMR), and independent hippocampal (*n* = 77) and peripheral blood (*n* = 141) cohorts. Five candidate neuroimmune hub genes (*TGFB1*, *ICAM1*, *TLR4*, *TLR2* and *CCL2*) were prioritized using a prespecified, threshold-dependent workflow and mapped to a microglia–cerebrovascular endothelium–peripheral myeloid cell axis. sc/snRNA-seq identified an activated endothelial subpopulation, detected only in TLE samples, and expansion of a microglial compartment with disease-associated microglia (DAM)-like features; CellChat and MultiNicheNet prioritized microglia-derived SPP1, C1QA and MIF as candidate mediators of endothelial inflammatory activation via S1PR1/ITGB1, CD93 and CD74. Plasma kynurenine showed a nominally significant association with focal epilepsy in MR (inverse-variance weighted [IVW] OR = 1.96, 95% CI 1.10–3.52, *p* = 0.024), whereas SMR did not detect genetically predicted effects of *TLR4* or *TLR2* expression on focal epilepsy risk; these findings do not establish an acquired disease mechanism. A *TGFB1* + *TLR4* two-gene signature showed exploratory discrimination in an independent hippocampal cohort (area under the receiver operating characteristic curve [AUC] = 0.868), and blood *TLR4* was selectively elevated in drug-naïve newly diagnosed patients (AUC = 0.684). Targeted gene set enrichment analysis (GSEA) and molecular docking nominated simvastatin as a repurposing candidate predicted computationally to interact with MD-2; this prediction has not been functionally validated. This multi-cohort study identifies a TLR4-associated microglia–endothelial inflammatory axis in drug-resistant TLE and generates hypotheses for prospective biomarker studies and experimental drug-repurposing research.

## 1. Introduction

Temporal lobe epilepsy (TLE) is the most common focal epilepsy in adults. Drug-resistant epilepsy is defined by failure of adequate trials of two tolerated, appropriately chosen and used anti-seizure medication schedules, whether as monotherapies or in combination, to achieve sustained seizure freedom [1]. Between 30% and 40% of patients with TLE fail to respond to anti-seizure medication [2], and resistance rates are highest in those with hippocampal sclerosis [3,4]. Even after anterior temporal lobectomy, seizure freedom is not guaranteed [5]. Clarifying the mechanisms of drug resistance and identifying non-invasive biomarkers and actionable targets therefore remain pressing clinical problems.

Neuroinflammation is a key pathological process driving epileptogenesis and the development of drug resistance [6,7,8,9]. Drug-resistant TLE is accompanied by sustained glial activation and pro-inflammatory cytokine release [10]; experimental and clinical evidence also implicates blood–brain barrier dysfunction and leukocyte–endothelial adhesion in epilepsy [11,12]. In animal models, HMGB1 acting through TLR4 and IL-1β acting through IL-1R1 both enhance neuronal excitability and promote seizure activity [13,14], and the CCL2–CCR2 axis mediates central monocyte infiltration [15]. Most of this work has addressed single molecules, and an integrated characterization of the immune regulatory network in drug-resistant TLE is still lacking. It also remains incompletely understood whether the observed expression changes reflect acquired disease processes or inherited genetic susceptibility, and how central and peripheral immune signals are compartmentalized [16]. Gut microbiota and their metabolites, including kynurenine, can influence neuroinflammation and blood–brain barrier function [17,18,19,20,21], but their causal relationships with epilepsy remain incompletely defined. Most currently available anti-seizure medications primarily target neuronal excitability, whereas epidemiological data linking statin use to a reduced risk of epilepsy [22,23] have motivated interest in drug repurposing.

Here we integrate a paired hippocampal transcriptome of drug-resistant TLE, an integrated temporal-lobe single-cell/nucleus transcriptome, large-scale genome-wide association study (GWAS) summary data and several independent validation cohorts. We prioritized candidate neuroimmune hub genes, characterized inferred microglia–vascular endothelial communication at single-cell/nucleus resolution, explored potential causal associations of gut-derived metabolites using Mendelian randomization, and evaluated peripheral expression, diagnostic performance and potential repurposing opportunities.

## 2. Results

### 2.1. Transcriptional Reprogramming of the Drug-Resistant TLE Hippocampus and Prioritization of Five Candidate Neuroimmune Hub Genes

Paired DESeq2 analysis of the discovery cohort identified 2749 differentially expressed genes (DEGs; 1394 upregulated and 1355 downregulated; false discovery rate [FDR] < 0.05; Supplementary Figure S1). Because this paired design compares sclerotic hippocampus with autologous temporal neocortex, the resulting DEGs may reflect both intrinsic brain-region differences and disease-related changes; stepwise cross-cohort convergence was therefore used to prioritize disease-associated signals. We first applied the same thresholds in the independent cohort GSE256068 (hippocampus from patients with TLE and hippocampal sclerosis [TLE-HS], *n* = 64, versus normal hippocampus, *n* = 13), which identified 2660 DEGs. Among 15,423 genes detected in both cohorts, 838 were differentially expressed in both, accounting for 30.5% of the discovery-cohort DEGs (hypergeometric *p* = 9.2 × 10^−86^; Supplementary Figure S9), and 527 of these (62.9%) were directionally concordant (binomial *p* = 4.1 × 10^−14^). The hub genes *TLR4* (log_2_FC = 1.29, FDR = 1.8 × 10^−7^) and *CCL2* (log_2_FC = 1.87, FDR = 4.3 × 10^−3^) were shared, directionally concordant DEGs, and *TGFB1* was also significantly upregulated (FDR = 5.1 × 10^−3^). The core neuroimmune signal is therefore enriched for disease-associated changes, although regional sampling effects cannot be excluded.

**Figure 1:**
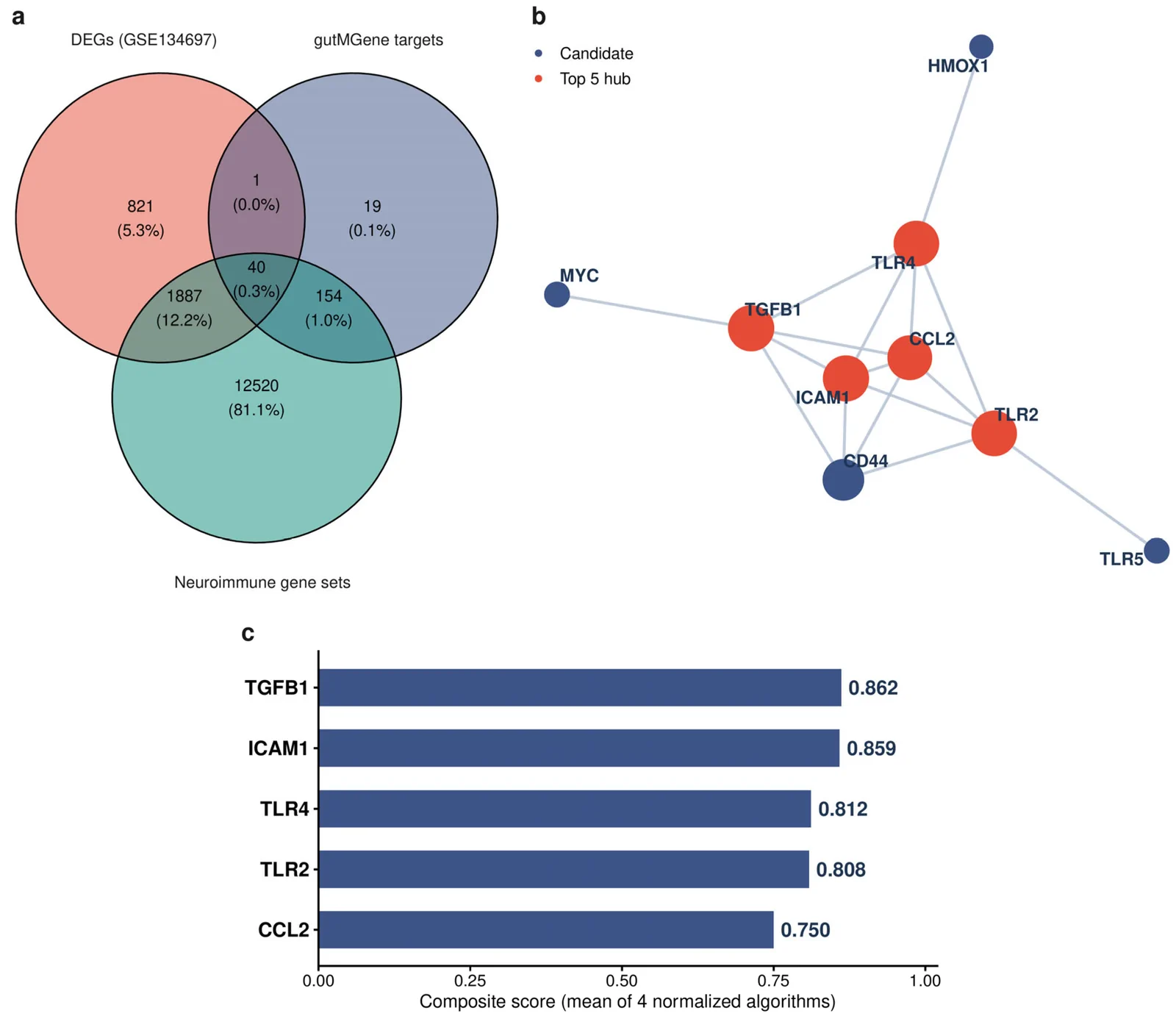
Screening and prioritization of candidate neuroimmune hub genes in the drug-resistant TLE hippocampus. (**a**) Venn diagram of the triple intersection among differentially expressed genes (paired DESeq2 in GSE134697), gut-microbe/metabolite target genes (gutMGene), and MSigDB neuroimmune gene sets. (**b**) STRING protein–protein interaction network of prioritized candidate genes, with the top five hub genes highlighted. (**c**) Composite cytoHubba scores for the five hub genes.

The DEGs were next intersected with gutMGene gut-microbe and metabolite target genes and with MSigDB neuroimmune gene sets. This triple intersection, used as a candidate prioritization strategy rather than a mechanistic presumption, yielded 40 primary candidate genes (Figure 1a) spanning innate immune molecules such as *TNF*, *IL1B*, *CCL2*, *TGFB1* and *TLR2/4/5/8*. Weighted gene co-expression network analysis (WGCNA; power = 14, signed R^2^ ≈ 0.80) identified 9 modules, of which the yellow module (651 genes) showed the strongest correlation with sclerotic hippocampus (r = 0.80, *p* = 2 × 10^−8^; Supplementary Figure S2); intersection of these candidates with disease-associated modules yielded 15 high-priority genes. An integrated score across four cytoHubba algorithms applied to the STRING network prioritized five candidate hub genes: *TGFB1* (0.862), *ICAM1* (0.859), *TLR4* (0.812), *TLR2* (0.808) and *CCL2* (0.750) (Figure 1b,c; Supplementary Table S2). These genes were functionally enriched in inflammation-related processes including phagocytosis, glial cell differentiation and regulation of chemokine and IL-6 production. A revision-stage sensitivity analysis conditional on the nine genes mapped in the primary STRING network assessed combined-score thresholds of 400, 550, 700, 850 and 900. At the primary threshold of 700, MCC, Degree and EPC each returned the original five genes in the top five, whereas BottleNeck substituted *CD44* for *ICAM1*. Across thresholds of 400–850, *TGFB1*, *TLR4*, *TLR2* and *CCL2* remained in the composite top five, whereas *ICAM1* dropped out at thresholds of 550 and 850. At the extremely stringent threshold of 900, the network retained only four edges and *TGFB1* dropped out of the composite top five. Thus, the analysis supports these genes as prioritized candidates under the prespecified workflow, but their ranks are not invariant to network sparsity or ranking method.

### 2.2. Regulatory Provenance and Cellular Localization of Hub Genes

gutMGene tracing matched the five hub genes to 24 experimentally validated microbe–metabolite–gene regulatory records (Supplementary Table S5). Butyrate suppresses *CCL2* and *TLR4*, succinate activates *CCL2* and propionate activates *TLR2*; *Akkermansia muciniphila* and related strains regulate *TLR4* and *TLR2*; *Faecalibacterium prausnitzii* and *Escherichia coli* Nissle 1917 activate *TGFB1*; and phenolic acid metabolites activate *ICAM1*. These relationships derive from in vitro and animal evidence and require separate testing in human TLE. Human Protein Atlas (HPA) immunohistochemistry showed that the encoded proteins were generally undetectable or present at very low levels in normal human hippocampal tissue, with only CCL2 showing low-to-moderate staining. This contrasts with their upregulation in the discovery TLE hippocampus and is compatible with disease-associated transcriptional upregulation. Single-cell-type expression profiling assigned the expression patterns of these five genes predominantly to three compartments: brain microglia, vascular endothelial cells and peripheral monocytes/macrophages (Supplementary Figure S3).

### 2.3. Integrated Single-Cell/Nucleus Transcriptome Atlas and Cellular Composition Remodeling

**Figure 2:**
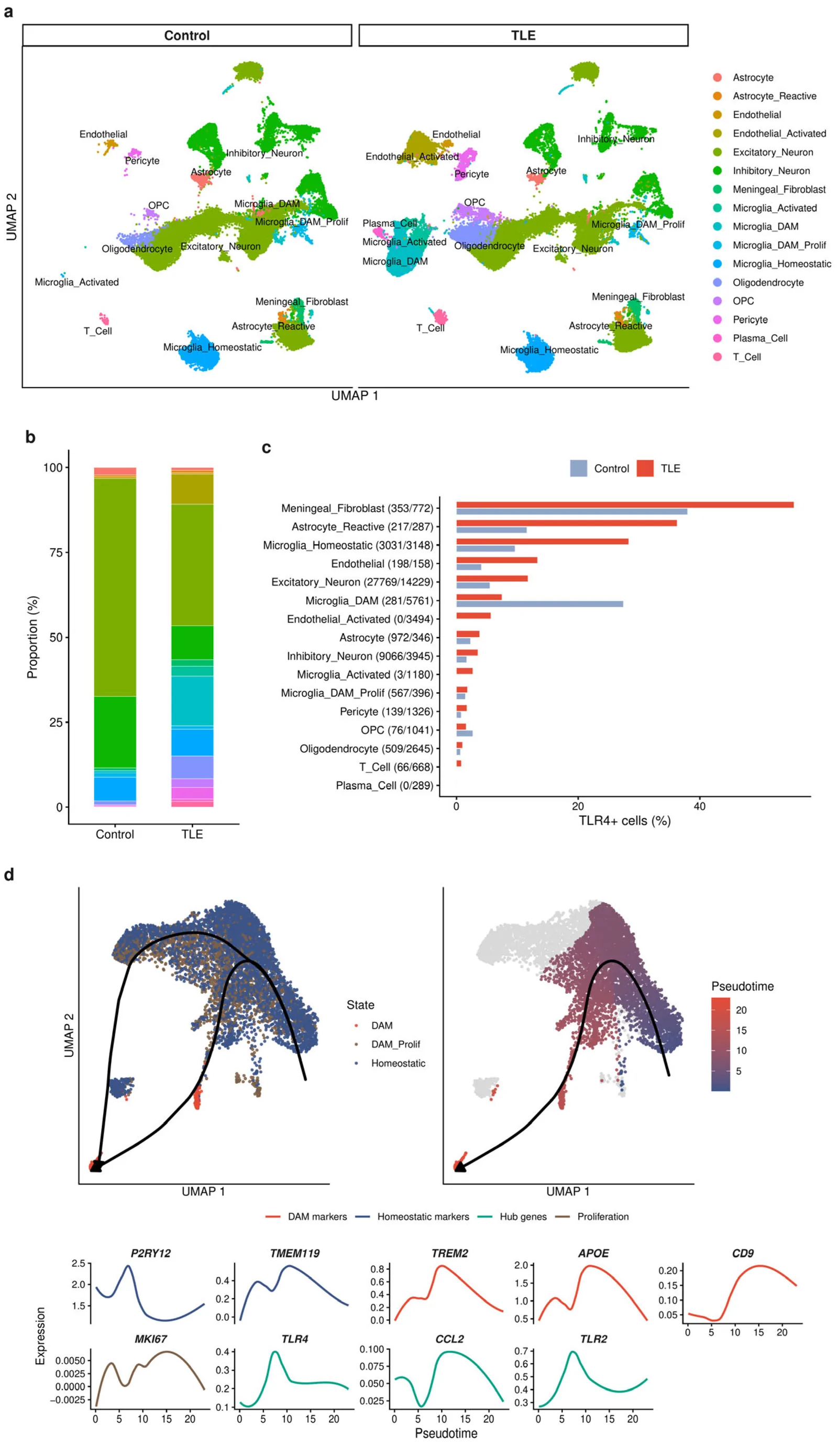
Integrated single-cell/nucleus transcriptome atlas and cellular composition remodeling in drug-resistant TLE. (**a**) UMAP of 82,932 quality-filtered transcriptomes (16 cell types, split by control/TLE). (**b**) Stacked bar plots of cell-type proportions. (**c**) TLR4-positive cell proportions across cell types. (**d**) Slingshot pseudotime trajectories in the microglial subset and expression dynamics of selected markers and hub genes.

Integrated sc/snRNA-seq of temporal-lobe samples from 9 patients with TLE and 4 controls yielded 82,932 quality-filtered transcriptomes (43,247 control and 39,685 TLE). Donor-colored UMAPs showed visually reduced batch-associated clustering after Harmony integration, although this visual assessment does not establish complete removal of batch effects (Figure 2a). In the initial annotation, microglial clusters co-expressed the plasma-cell markers *JCHAIN*/*MZB1*/*IGKC*; after marker-based dual filtering of putative ambient-RNA contamination (Supplementary Figure S4), the atlas comprised 16 cell types (Supplementary Figure S5). Individual-level comparisons (Figure 2b) showed a 55% reduction in the proportion of excitatory neurons (*p* = 0.028), consistent with the pathology of hippocampal sclerosis, and an activated endothelial subpopulation detected only in TLE samples in this cohort (*p* < 0.001). This observation is consistent with endothelial inflammatory activation, but neither demonstrates increased blood–brain barrier permeability nor establishes a TLE-exclusive cell state, because limited control sampling and donor heterogeneity can affect detection of rare populations. The DAM-like microglial proportion (labeled DAM in the figures for brevity) showed a 38.5-fold numerical increase in individual-level comparisons that did not reach individual-level significance, whereas the oligodendrocyte precursor cell (OPC) proportion increased 15.0-fold (*p* = 0.024). Plasma cells accounted for only 0.7% of TLE cells, indicating low plasma-cell abundance in this dataset.

Milo neighborhood testing and pseudobulk analysis provided complementary support for these findings. Neighborhoods of DAM, activated endothelium, and OPCs were significantly enriched and excitatory neurons significantly depleted in TLE (FDR < 0.05), and the hub genes *TLR4*, *TGFB1*, *CCL2* and *ICAM1* were all significantly upregulated. These sample-level analyses reduce the risk of spurious findings from single-cell pseudoreplication but do not eliminate sampling uncertainty. Across the atlas, microglia encompassed four states: homeostatic, activated, proliferating DAM and DAM. Slingshot pseudotime analysis of the microglial subset reconstructed two activation trajectories originating from homeostatic microglia and leading to proliferating DAM and to DAM, respectively (Figure 2d), with *TLR4*/*TLR2* upregulated along the DAM trajectory. Control and TLE cells were distributed along both trajectories, suggesting that TLE mainly amplifies the DAM compartment quantitatively rather than inducing an entirely new state.

Analysis of TLR4-positive cell proportions (Figure 2c) showed that the baseline positivity rate was highest in meningeal fibroblasts (38.0% in controls, reflecting constitutive expression), whereas TLE-associated increases were concentrated on the microglia–endothelium axis: homeostatic microglia rose from 9.6% to 28.3% and endothelium from 4.0% to 13.3%. The activated microglial and endothelial subpopulations detected only in TLE samples contained 2.6% and 5.6% TLR4 + cells, respectively. Control DAM showed a 27.4% positivity rate, but this subset contained only 281 cells, and the rate fell to 7.4% after the approximately 20-fold DAM expansion in TLE. Given the small cell numbers, this pattern should be interpreted cautiously and not as evidence of a directional decrease.

### 2.4. Cell-Type-Specific Pathological Programs

Pseudobulk differential analysis showed systematic downregulation of core epilepsy genes in excitatory neurons (*SCN1A*, *SCN2A*, *GRIA1*, *GRIN2B*, *GABRB2* and *GABRB3*). Reactive astrocytes showed upregulation of *B2M*, *APOE*, *GJA1* and *AQP4* accompanied by *SLC1A2* (EAAT2) downregulation, consistent with altered immune-related and homeostatic functions, including potentially impaired glutamate clearance [24,25]. These markers were therefore interpreted as evidence of a context-dependent reactive state rather than a discrete A1/A2 classification. Microglia exhibited DAM-like features, with high expression of *TREM2*, *APOE*, *TYROBP* and HLA-DR [26]. These markers support disease-associated reactivity but do not by themselves establish the full DAM phenotype. We therefore use DAM-like as an operational annotation; systematic assessment of homeostatic, phagocytic, lysosomal and lipid-associated programs, together with functional validation, is required to establish DAM identity and its biological role in TLE. Cross-cell-type comparison identified 646 genes differentially expressed in both excitatory neurons and microglia, of which 640 (>99%) were directionally concordant and enriched in cytoplasmic translation and ribosome biogenesis. This is consistent with disruption of translation- and oxidative-phosphorylation-related programs, although mTOR-pathway involvement remains hypothetical.

### 2.5. Intercellular Communication Within the Microglia–Vascular Endothelial Inflammatory Axis

**Figure 3:**
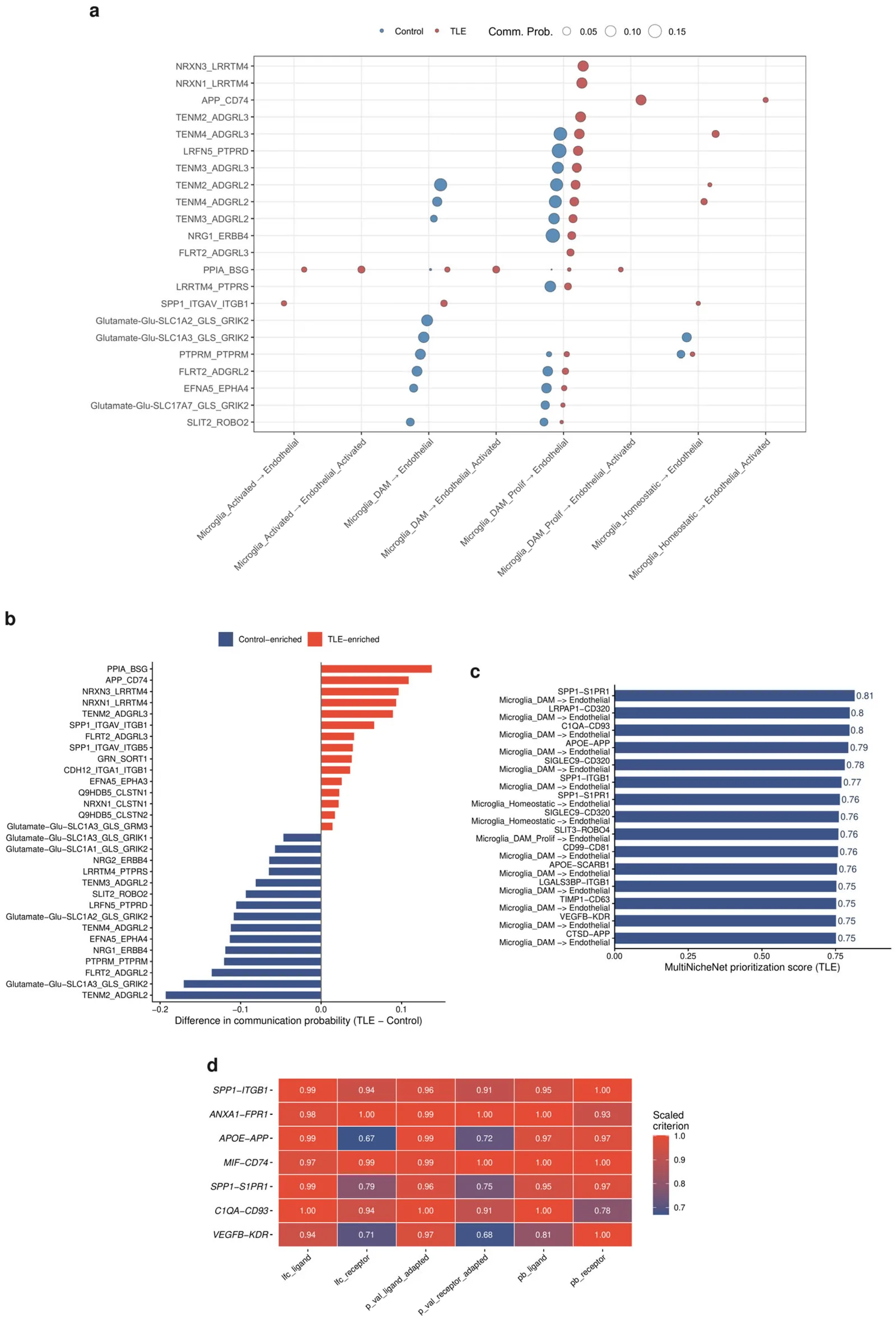
Intercellular communication within the microglia–vascular endothelial inflammatory axis. (**a**) CellChat-inferred microglia→endothelium ligand–receptor interactions compared between control and TLE. (**b**) Between-group differences in CellChat communication probability for microglia–endothelial signaling. (**c**) MultiNicheNet prioritization scores for selected microglia–endothelial interactions in TLE. (**d**) Scaled MultiNicheNet prioritization criteria for selected ligand–receptor pairs.

CellChat between-group comparison showed that the number of inferred interactions in TLE increased by 51.3% relative to controls (from 7726 to 11,691 pairs), whereas total interaction strength decreased by 21.8%. This pattern is consistent with altered communication homeostasis: synaptic adhesion and neurotransmission signals that dominated in controls receded in TLE, while inflammatory pathways including COMPLEMENT, MIF and MHC-II were detected only in TLE. The microglia–endothelium axis shifted from synaptic adhesion signals in controls to APP-CD74, PPIA-BSG and SPP1-(ITGAV + ITGB1) signaling in TLE, and TGFB1-(TGFBR1 + TGFBR2) was detected only in TLE and was confined to the microglial lineage (Figure 3a,b).

MultiNicheNet multi-sample analysis provided sample-level support for these differences. Microglia_DAM was the most transcriptionally perturbed cell type in TLE (3315 differential genes), and glial cells (5932) and vascular-associated cells (2900) were far more perturbed than neurons (831). Among immune interactions, Microglia_DAM-to-pericyte SPP1-ITGB1 ranked 9th overall and was detected in all 9 TLE samples, and T cell-to-Microglia_DAM ANXA1-FPR1 entered the top 20 (Figure 3d). The highest-priority microglia–endothelium interactions were SPP1-S1PR1, C1QA-CD93, APOE-APP and VEGFB-KDR (Figure 3c), and LY86 (MD-1), a regulator of TLR4-related signaling, ranked first in ligand activity analysis. On the receiving side, activated endothelium highly expressed *CLDN5* and *ICAM1*/*VCAM1*/*SELE*, with upregulated genes enriched in leukocyte migration and tight junction regulation. This transcriptional pattern is compatible with possible peripheral immune cell extravasation via ICAM1-ITGAL/ITGB2, but does not demonstrate extravasation. Bulk deconvolution with MCP-counter and quanTIseq consistently showed increased myeloid fractions, decreased natural killer (NK) cell fractions and higher inferred M2-like macrophage scores in the TLE hippocampus, concordant with the single-cell landscape. These scores describe a transcriptional state within a continuous activation spectrum and should not be interpreted as evidence of a discrete M1/M2 polarization endpoint [27].

### 2.6. Genetic Association Between Plasma Kynurenine and Epilepsy, and Genetic Attribution of Hub Genes

**Figure 4:**
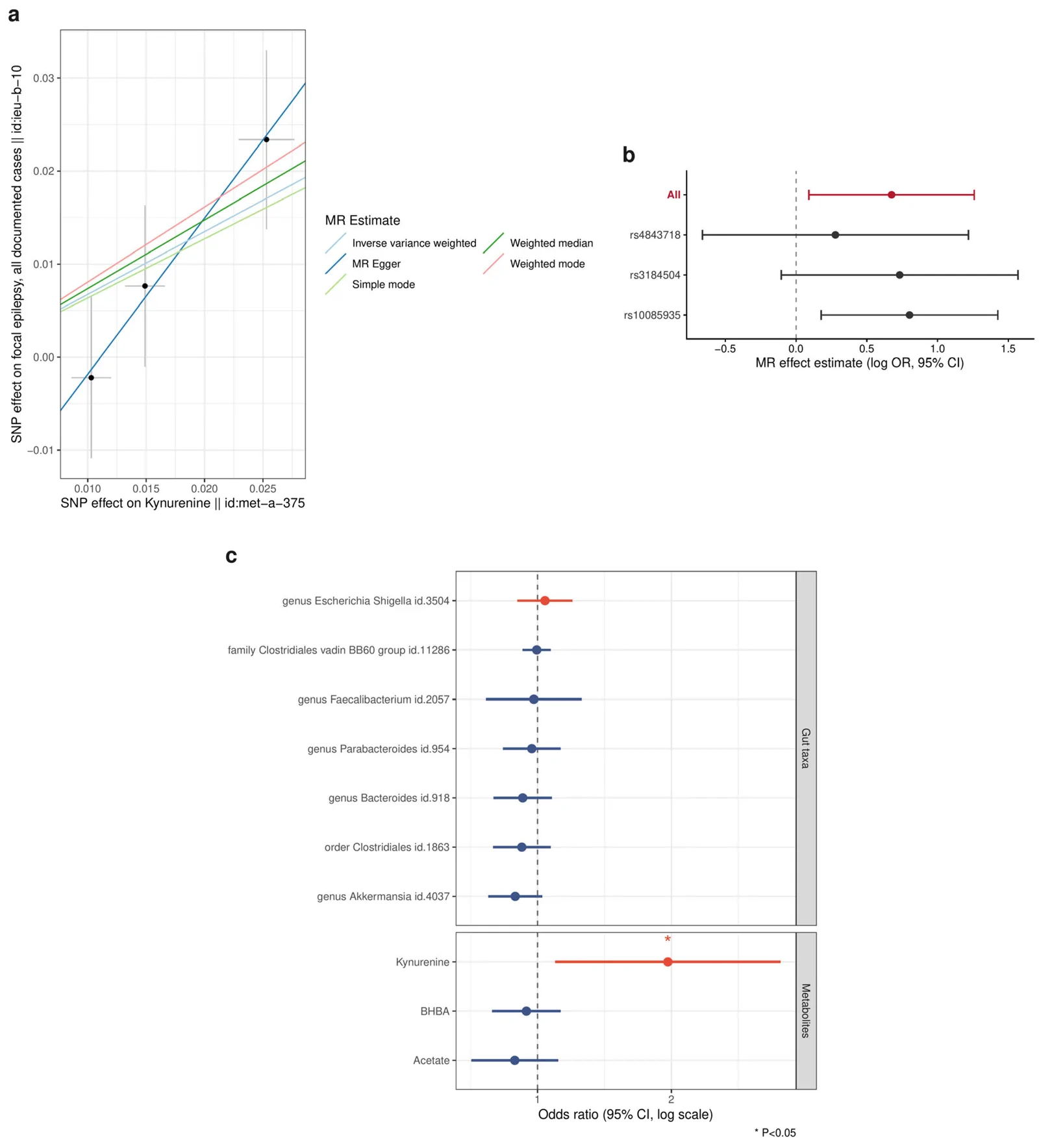
Two-sample Mendelian randomization of plasma kynurenine and focal epilepsy. (**a**) MR scatter plot of the three genome-wide significant SNPs with fitted effect lines (IVW: OR = 1.96, 95% CI 1.10–3.52, *p* = 0.024). (**b**) Leave-one-out analysis of the kynurenine–focal epilepsy MR association. (**c**) Forest plot of selected exploratory gut microbiota and metabolite MR associations.

Two-sample MR showed that plasma kynurenine levels were positively associated with focal epilepsy risk (IVW: OR = 1.96, 95% CI 1.10–3.52, *p* = 0.024, nominally significant; Figure 4a; Supplementary Table S3). The analysis included 3 genome-wide significant SNPs with a mean F statistic of 114.42. Cochran’s Q test did not detect heterogeneity (*p* = 0.445), and the MR-Egger intercept did not detect directional horizontal pleiotropy (*p* = 0.427); however, power for these sensitivity analyses was limited by the small number of instruments. Leave-one-out analysis was directionally consistent (Figure 4b). In the exploratory microbiome scan, 9 of 203 taxa reached nominal significance, none of which remained statistically significant after Benjamini–Hochberg (BH) correction (Supplementary Figure S6), and reverse MR did not detect an association in the opposite direction. In SMR analysis, only *TLR4* and *TLR2* among the five hub genes had eligible cis-eQTL instruments, and neither showed a significant association between expression and focal epilepsy risk (*TLR4*: b = 0.003, *p* = 0.89; *TLR2*: b = 0.024, *p* = 0.40). One possible explanation for their hippocampal upregulation is an acquired, disease-associated change, consistent with the low homeostatic expression seen in HPA; however, this negative SMR result does not establish that interpretation or exclude genetic effects.

### 2.7. Cross-Cohort Validation, Peripheral Detectability and Diagnostic Signature

**Figure 5:**
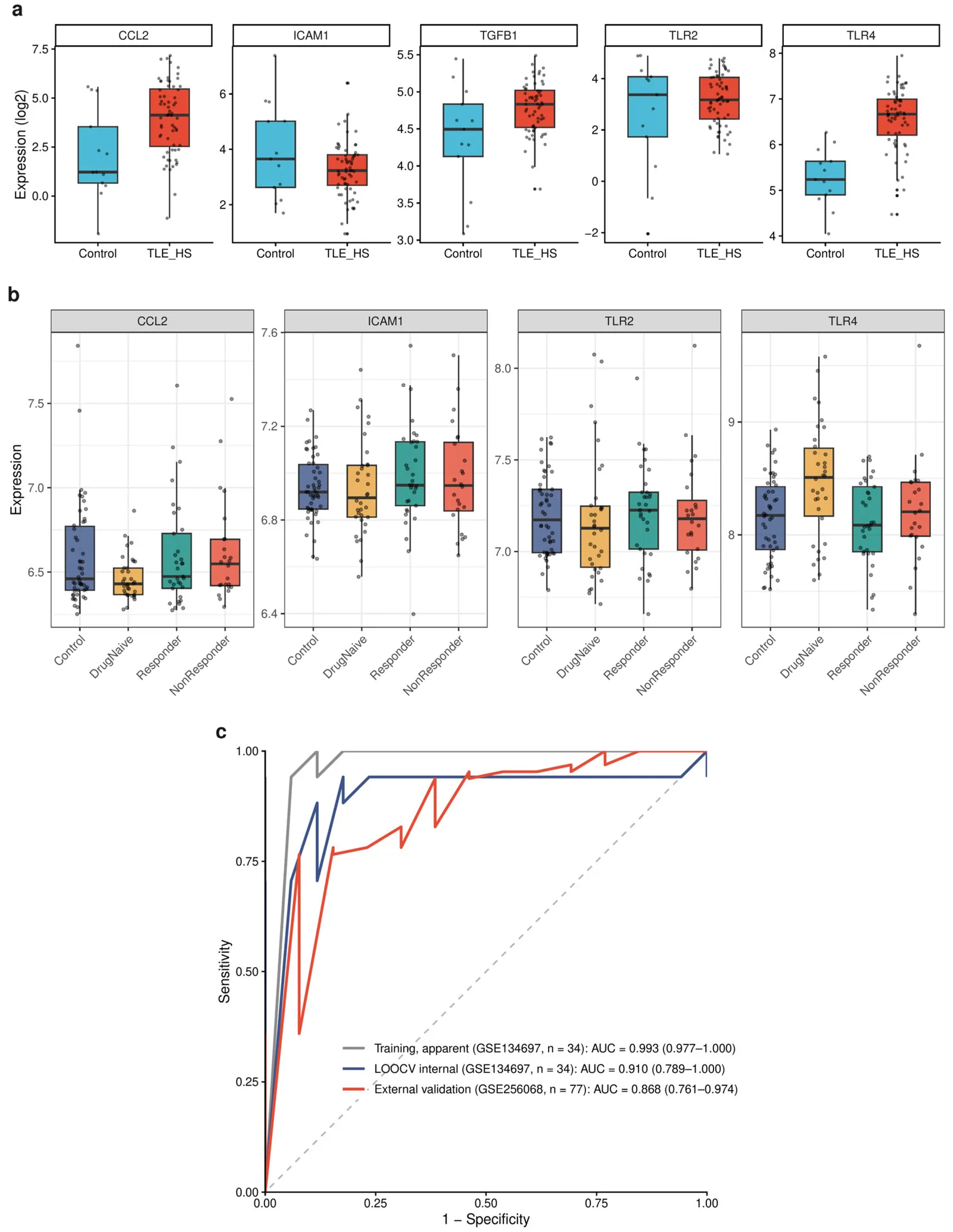
Cross-cohort validation of hub genes and diagnostic performance. (**a**) Expression of the five hub genes in the external hippocampal cohort GSE256068 (TLE-HS, *n* = 64 vs. normal hippocampus, *n* = 13). (**b**) Peripheral blood expression of *CCL2*, *ICAM1*, *TLR2* and *TLR4* in healthy controls, drug-naïve newly diagnosed patients, drug-responsive patients and drug-resistant patients. (**c**) ROC curves for the *TGFB1* + *TLR4* signature in the training cohort, LOOCV internal validation and external validation (external AUC = 0.868). The dashed diagonal line indicates the reference line for a random classifier (AUC = 0.5).

In the external hippocampal cohort GSE256068, *TGFB1*, *TLR4* and *CCL2* were all significantly upregulated with directions concordant with the discovery cohort. *TLR2* showed a concordant trend that did not reach significance with only 13 controls, *ICAM1* was not replicated, and the *TLR4* effect size exceeded that of the discovery cohort (Figure 5a). In the peripheral blood cohort GSE143272, *TLR4* was significantly elevated only in drug-naïve newly diagnosed patients (logFC = 0.33, *p* = 6.4 × 10^−4^, FDR = 0.008) and was not significantly elevated in the treated patient groups (Figure 5b). This pattern is compatible with evidence that valproate can alter peripheral inflammatory gene expression [28], although the cross-sectional design cannot establish treatment effects. It also suggests central–peripheral compartmentalization of the inflammatory signal. Peripheral blood *TLR4* distinguished drug-naïve patients from healthy controls with an AUC of 0.684 (95% CI 0.573–0.813), while all other hub genes had AUCs below 0.61.

Using the five hub genes as the candidate pool, least absolute shrinkage and selection operator (LASSO) and support vector machine recursive feature elimination (SVM-RFE) were intersected to define a *TGFB1* + *TLR4* two-gene signature with similar model coefficients. The apparent training-cohort AUC was 0.993 (95% CI 0.977–1.000), with 94.1% sensitivity and 100% specificity at the prespecified threshold of 0.48; given the small training set of only 17 pairs, the model should be regarded as exploratory. Leave-one-out cross-validation (LOOCV) internal validation of the fixed signature gave an AUC of 0.910 (95% CI 0.789–1.000), with sensitivity, specificity and accuracy all 88.2% at the threshold of 0.48 (Supplementary Figure S8), although feature-selection optimism and sampling variability remain important concerns. The AUC in the external hippocampal cohort was 0.868 (95% CI 0.761–0.974), with 67.2% sensitivity, 92.3% specificity and 71.4% accuracy (Figure 5c; Supplementary Table S4). Peripheral blood *TLR4* alone gave an AUC of 0.684 (Supplementary Table S4).

### 2.8. Predicted Effects of TLR4 Intervention and Candidate Drug Repurposing

**Figure 6:**
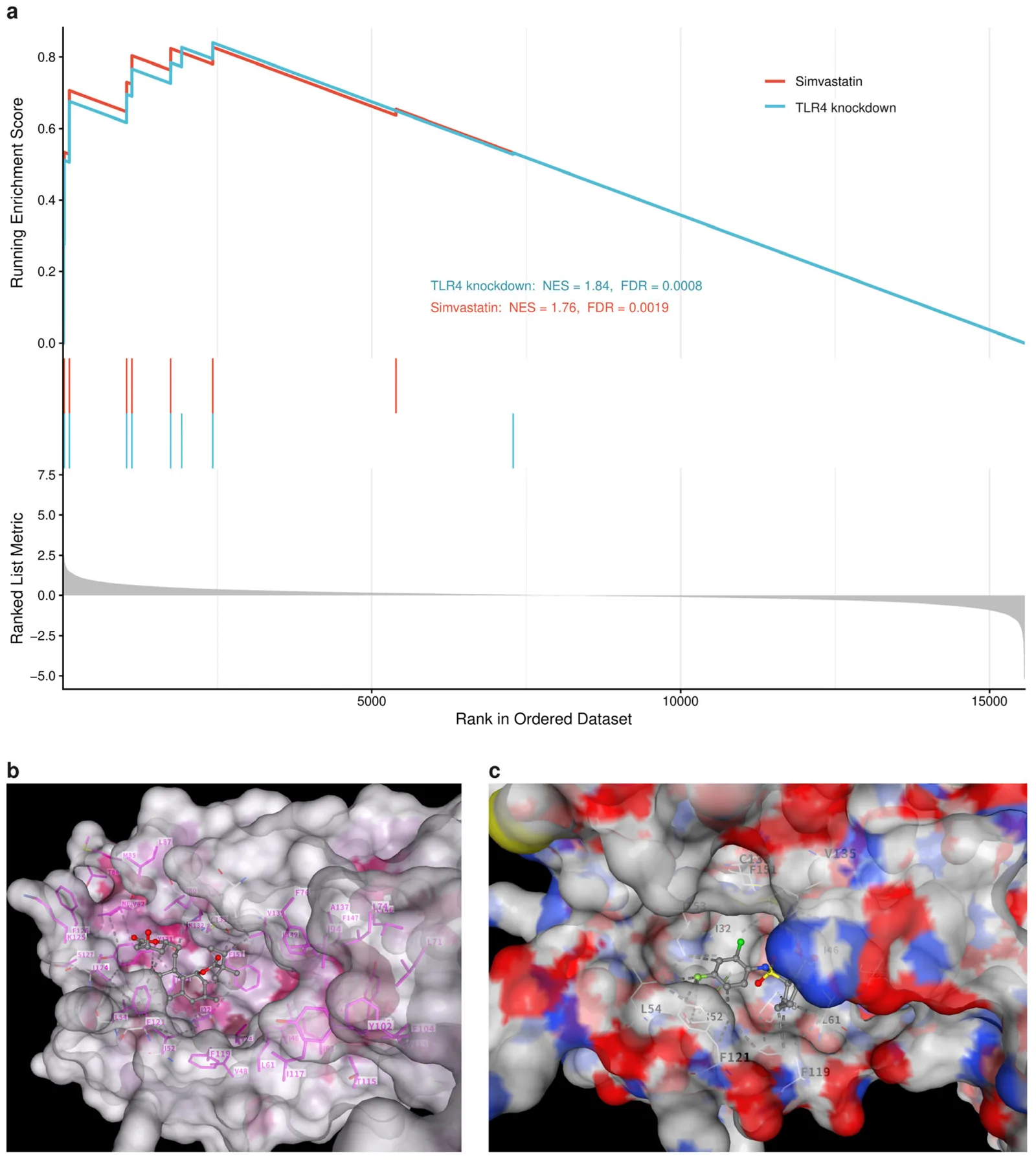
Predicted effects of TLR4 intervention and candidate drug repurposing. (**a**) Targeted GSEA enrichment curves of the TLR4-knockdown and simvastatin response gene panels. (**b**) Overall surface view of the predicted simvastatin docking pose in the MD-2 hydrophobic pocket. (**c**) Close-up view of the predicted pose and surrounding residues. In (**a**), the red and cyan curves represent the simvastatin and TLR4-knockdown response gene sets, respectively; vertical ticks indicate gene-set members, and the gray filled curve shows the ranked-list metric. In (**b**,**c**), the molecular surface, binding-site residues, and simvastatin are shown in contrasting colors; magenta sticks indicate surrounding residues, and the ligand is shown with standard atom coloring.

Targeted GSEA showed that the *TLR4*-knockdown response gene panel was significantly enriched among genes upregulated in the TLE hippocampus (NES = 1.84, FDR = 0.0008), as was the simvastatin response panel (NES = 1.76, FDR = 0.0019) (Figure 6a). Both enrichments motivated the hypothesis that these perturbations might modulate the TLE inflammatory transcriptional signature, although directionality cannot be established from these analyses alone. Together with the sustained upregulation of *TLR4*/*TLR2* along the DAM activation trajectory (Figure 2d), they support TLR4 as a candidate upstream regulatory node of the microglial inflammatory axis. In DSigDB dual-input enrichment analysis, simvastatin was significantly enriched with both inputs (hub-gene input FDR = 3.7 × 10^−7^, ranking first; upregulated-DEG input FDR = 3.4 × 10^−3^; Supplementary Figure S10). The most favorable docking score for simvastatin was −8.2 kcal/mol, with the predicted simvastatin pose occupying the MD-2 hydrophobic ligand pocket, the known binding site of TLR4 antagonists (Figure 6b,c). TAK-242 yielded a docking score of −7.8 kcal/mol, but this value is not a positive-control benchmark because TAK-242 acts at an intracellular TLR4 site rather than the MD-2 pocket [29]. Among transcripts related to *TLR4* signaling, TLR4 (log_2_FC = 1.17 in homeostatic microglia, FDR = 2.3 × 10^−65^), the co-receptor *LY96* (log_2_FC = 5.38 in DAM, FDR = 1.3 × 10^−12^) and the downstream effector *TNF* (log_2_FC = 5.46 in DAM, FDR = 3.9 × 10^−6^) were significantly upregulated in the TLE microglial compartment (Supplementary Figure S11). Simvastatin has an established clinical use, but direct TLR4/MD-2 binding, pathway inhibition and anti-seizure efficacy remain unverified and require experimental testing.

## 3. Discussion

We prioritized five candidate neuroimmune hub genes in drug-resistant TLE, namely, *TGFB1*, *ICAM1*, *TLR4*, *TLR2* and *CCL2*, using a threshold-dependent workflow, and mapped them onto a candidate microglia–cerebrovascular endothelium–peripheral myeloid cell inflammatory axis. Milo and pseudobulk analyses supported enrichment of an activated endothelial subpopulation and a DAM-like compartment in the TLE hippocampus while reducing pseudoreplication-related bias. Two independent methods, CellChat and MultiNicheNet, prioritized microglia-derived ligands including SPP1, C1QA and MIF as candidate mediators of endothelial inflammatory activation via the S1PR1/ITGB1, CD93 and CD74 receptors. MR yielded a nominal association between plasma kynurenine and focal epilepsy, and SMR did not detect genetically predicted effects of *TLR4* or *TLR2* expression; neither result establishes the causal sequence of the proposed axis. At the translational level, perturbation analysis raised the hypothesis that *TLR4* knockdown and simvastatin might modulate the TLE inflammatory transcriptional signature, although the direction of any such effect cannot be determined from enrichment alone.

Our initial microglial annotation was complicated by co-expression of plasma-cell markers; after dual filtering, plasma cells accounted for only 0.7% of TLE cells. This finding indicates limited plasma-cell abundance in the present dataset but does not exclude adaptive immune infiltration in other cohorts. Failure to account for ambient immunoglobulin RNA may overestimate plasma-cell-related signals in single-nucleus data. Relative to the DAM state originally defined in neurodegeneration [26], expansion of the DAM-like compartment in TLE appears to represent quantitative amplification rather than an entirely new state [30], although full phenotypic equivalence has not been established. The concurrent presence of an activated endothelial subpopulation, however, suggests that microglial activation and vascular inflammation are coupled in epilepsy. The upregulation of *TLR4*/*TLR2* along the activation trajectory, together with the significant enrichment of the TLR4-knockdown response panel among TLE-upregulated genes, prioritizes TLR4 for functional testing as a candidate upstream regulatory node [27,31].

Integrating our findings with prior evidence, a candidate mechanistic axis can be proposed: altered gut-derived metabolite signaling → microglial TLR2/4-associated activation → expansion of a DAM-like state → release of SPP1, C1QA and MIF → endothelial and pericyte receptor signaling with adhesion molecule upregulation → potential blood–brain barrier dysfunction → excitatory neuron loss. The strength of evidence differs across links. The kynurenine–epilepsy association is a nominal MR finding from this work, but any direct effect of kynurenine on microglial activation is a literature-based inference. Microglial TLR2/4-associated activation and DAM-like conversion are transcriptomic observations, not functional demonstrations of TLR2/4 dependence. The ligand–receptor axis is supported by two computational communication methods, and endothelial adhesion molecule upregulation is directly observed at the transcript level; however, neither demonstrates increased blood–brain barrier permeability. The contribution of blood–brain barrier dysfunction to TLE progression is supported by prior literature [32], while the causal ordering of inflammation, barrier dysfunction and neuron loss cannot be established from our data. The upstream link from gut-derived metabolites to microglial activation carries the weakest evidence and, together with the terminal link, constitutes a priority direction for experimental validation. In particular, no microbiome MR association survived BH correction; accordingly, the microbiome MR results do not support the proposed mechanistic sequence, and the gutMGene links are retained solely as hypotheses derived from experimental contexts outside the present human TLE cohorts.

Kynurenine also requires pathway-level rather than single-metabolite interpretation. It is an intermediate of tryptophan metabolism, and downstream metabolites can exert distinct or opposing effects on neuronal excitability and neuroinflammation [20]. Accordingly, the nominal MR association should not be interpreted as direct activation of TLR4 signaling by kynurenine; it could reflect altered pathway flux or correlated downstream metabolites. IDO1, IDO2, TDO2, KMO and KYNU are plausible regulators of this pathway, but the present analyses do not establish their differential activity or involvement in TLE. Integrated measurements of enzyme expression and activity, together with targeted metabolomics, will be needed to test this possibility.

Two considerations are relevant to biomarker development. First, peripheral blood TLR4 was significantly elevated only in drug-naïve newly diagnosed patients (AUC = 0.684) and was not significantly elevated in the treated groups, so its potential utility should be restricted to evaluation as an early adjunctive biomarker; it should not be extrapolated to drug-resistance stratification. Second, TLR4 expression has previously been reported in human epileptic brain tissue [33]. In our data, the juxtaposition of persistent central TLR4 overexpression in drug-resistant patients with the absence of significant peripheral elevation in treated groups is compatible with central–peripheral compartmentalization and motivates evaluation of central TLR4-directed interventions in patients already receiving medication, without implying established clinical benefit. The simvastatin repurposing hypothesis is motivated by indirect computational evidence (perturbation-response enrichment and a docking score of −8.2 kcal/mol) and epidemiological associations between statin use and reduced epilepsy risk [22,23]. Its established clinical use makes it tractable for further study, but direct TLR4/MD-2 binding, pathway inhibition and anti-seizure efficacy require experimental confirmation.

Several limitations should be noted. This study is a retrospective secondary analysis of public datasets, with no original in vitro or in vivo functional experiments. The paired discovery design compares sclerotic hippocampus with temporal neocortex within patients; brain region and disease-related hippocampal pathology therefore cannot be fully separated. Overlap analysis against the external normal-hippocampus cohort showed that 838 genes (30.5%) replicated with 62.9% directional concordance and that several candidate hub genes showed concordant directions, indicating enrichment for disease-associated changes rather than proving TLE specificity. The external cohort included only 13 controls, leaving regional and sampling effects incompletely resolved. Candidate prioritization also depends on the DEG, gene-set, WGCNA, STRING and ranking choices. The revision-stage sensitivity analysis tested STRING thresholds and cytoHubba methods conditional on the original nine-node network; WGCNA module assignments were not re-estimated, and invariance across module-selection criteria is therefore not claimed. The diagnostic model is exploratory and not clinically ready: LOOCV of the fixed signature yielded an AUC of 0.910, but optimistic bias at the feature-selection level cannot be fully excluded, and larger prospective cohorts with completely independent preprocessing and feature selection are required. Metabolite MR included only 3 SNPs and was nominally significant, no microbiome MR association survived multiple-testing correction, and the upstream links of the mechanistic axis remain exploratory hypotheses. SMR could test only TLR4 and TLR2 and did not detect significant associations, so an acquired expression mechanism remains an indirect interpretation rather than a demonstrated conclusion.

Additional limitations concern the integrated single-cell/nucleus analysis, pathway inference and docking. The cohort comprised only 9 TLE and 4 control donors, limiting power and making results susceptible to donor heterogeneity and unequal transcriptome yields. The detection of activated endothelium only in TLE samples may therefore reflect sampling or detection limits rather than a TLE-exclusive population. GSE190452 contributed nuclear RNA profiles from 4 TLE and 4 control donors on the NovaSeq 6000 platform, whereas GSE140393 contributed 5 additional TLE-only, unsorted single-cell RNA-seq samples on the HiSeq 4000 platform. Thus, preparation, platform, dataset and disease state are partially confounded, and Harmony cannot guarantee removal of these effects. Donor identity was modeled, but sex, age, seizure frequency, medication exposure and hippocampal-sclerosis status were unavailable as a complete harmonized covariate set and were not modeled. No dedicated doublet-removal algorithm or algorithmic ambient-RNA correction was applied, and post-Harmony diagnostics were visual rather than quantitative. Per-donor post-QC transcriptome counts were not archived in a machine-readable table in the original workflow, which is a further reporting limitation. CellChat and MultiNicheNet infer candidate communication from transcriptomic patterns; ligand–receptor co-expression does not establish functional signaling. The DAM-like annotation is based on selected disease-associated markers and does not establish the full homeostatic, phagocytic, lysosomal and lipid-associated DAM program. Endothelial adhesion-molecule expression indicates inflammatory activation but does not directly measure blood–brain barrier permeability. The kynurenine MR result does not identify pathway flux, downstream metabolites or the involvement of IDO1, IDO2, TDO2, KMO or KYNU. Perturbation-response enrichment and molecular docking are computational predictions; docking lacked random-compound controls, molecular-dynamics validation and experimental binding assays, and TAK-242 is not an appropriate positive control for the MD-2 pocket because of its distinct intracellular TLR4 binding mechanism. Simvastatin should therefore be viewed solely as a hypothesis-generating repurposing candidate, not as an evidence-based adjunctive treatment for epilepsy.

## 4. Materials and Methods

### 4.1. Data Sources

The following publicly available datasets were analyzed. GSE134697 served as the discovery cohort and provided paired bulk RNA-seq of sclerotic hippocampus and temporal neocortex from 17 patients with drug-resistant TLE [34]. GSE256068 provided an external hippocampal cohort of 64 TLE-HS cases and 13 normal hippocampal controls [35]. GSE143272 provided a peripheral blood cohort of 50 healthy controls, 34 drug-naïve newly diagnosed patients, 33 drug-responsive patients and 24 drug-resistant patients [36]. Genetic instruments were drawn from the MiBioGen microbiome GWAS (18,340 individuals) [37], the ILAE focal epilepsy GWAS (9671 cases and 29,677 controls) [38], and the Shin et al. blood metabolite GWAS collection in IEU OpenGWAS (effective kynurenine sample size 7816; ID: met-a-375) [39]. The integrated single-cell/nucleus transcriptome comprised temporal-lobe data from 9 patients with drug-resistant TLE and 4 controls, yielding 82,932 quality-filtered transcriptomes. GSE190452 contributed nuclear RNA profiles from 4 TLE and 4 non-epileptic control donors sequenced on the Illumina NovaSeq 6000 platform. GSE140393 contributed 5 additional TLE surgical samples of unsorted, freshly dissociated single-cell RNA-seq sequenced on the Illumina HiSeq 4000 platform. Consequently, the datasets differed in cellular preparation and sequencing platform, and dataset source was partially confounded with disease state because GSE140393 contributed only TLE samples. Dataset characteristics are summarized in Supplementary Table S1.

### 4.2. Differential Expression, Network Analysis and Hub Gene Screening

Bulk differential expression analysis used a DESeq2 paired model [40] with thresholds of |log_2_FC| > 0.58 and FDR < 0.05. A co-expression network was constructed with WGCNA, using a soft-thresholding power that satisfied scale-free R^2^ >0.8 [41]. Differentially expressed genes were intersected with gutMGene gut-microbe and metabolite target genes [42] and with MSigDB neuroimmune gene sets retrieved using msigdbr v7.5.1 [43], and the resulting triple intersection was then intersected with disease-associated modules. A protein–protein interaction network was built from STRING v12.0 (combined score ≥ 700) [44], and the five top-ranked genes according to an integrated score across four cytoHubba algorithms (MCC, Degree, BottleNeck and EPC) were taken as hub genes [45]. Enrichment analyses used clusterProfiler [46]. Protein localization was assessed using immunohistochemistry data from the Human Protein Atlas (HPA) at https://www.proteinatlas.org/ (accessed on 3 November 2026) [47] and brain single-cell-type transcriptome data. Immune cell fractions were estimated by deconvolution with MCP-counter and quanTIseq for cross-validation. Cross-cohort reproducibility was assessed in GSE256068 by comparing TLE-HS hippocampus (*n* = 64) with normal hippocampus (*n* = 13) using limma with trend adjustment and the same significance thresholds. Enrichment of the overlap between the differentially expressed genes of the two cohorts was evaluated by a hypergeometric test, and directional concordance by binomial test. For the revision-stage sensitivity analysis, the nine genes mapped in the primary STRING network were re-queried using STRING v12.0 at combined-score thresholds of 400, 550, 700, 850 and 900. MCC, Degree, BottleNeck and EPC rankings were recomputed using the same algorithmic definitions as the primary workflow, and the composite top-five sets were compared across thresholds. This analysis evaluated network-threshold and ranking-method stability conditional on the original mapped candidate set; it did not re-estimate WGCNA module assignments.

### 4.3. Integrated Single-Cell/Nucleus Transcriptome Analysis

The standard Seurat v4 workflow [48] was applied. Initial objects were created with min.features = 200 and min.cells = 3. Quality control retained transcriptomes with nFeature_RNA > 200 and <10,000, nCount_RNA > 200, mitochondrial reads < 10% and hemoglobin-gene reads < 1%; no ribosomal-gene threshold was applied. No dedicated doublet-removal algorithm was used. Microglia underwent marker-based dual filtering: cells were annotated by *P2RY12*/*TMEM119*/*CX3CR1*, and *JCHAIN*/*MZB1* double-positive cells were excluded. This filtering addressed putative ambient immunoglobulin RNA but was not an algorithmic ambient-RNA correction. Harmony v1.2.4 [49] was applied using donor identity (orig.ident) as the grouping variable, followed by clustering on the top 40 principal components at resolution = 0.6 and manual annotation using canonical markers. Batch diagnostics comprised donor-colored UMAPs before and after Harmony and QC violin plots; no quantitative batch-mixing metric was calculated. The total post-QC count was 82,932 transcriptomes, but per-donor post-QC counts were not archived as a machine-readable table in the original workflow and are therefore not reported. Donor identity was retained for sample-level analyses. Sex, age, seizure frequency, medication exposure and hippocampal-sclerosis status were not available as a complete harmonized covariate set across the two GEO datasets and were not included in the models. Pseudotime analysis used Slingshot [50] with homeostatic microglia as the starting cluster. Cell proportions were compared per individual by Wilcoxon test, and Milo v1.6.0 neighborhood differential abundance testing was used to evaluate cellular abundance while accounting for sample-level replication [51]. Communication analysis used CellChat v2 (triMean, with interactions involving fewer than 10 cells filtered out) [52] and MultiNicheNet v2.1.0 (pseudobulk of 13 samples, muscat/edgeR differential testing and multi-criteria prioritization, without additional batch or clinical covariates) [53,54,55]. Both communication methods were treated as inferential prioritization rather than evidence of functional signaling.

### 4.4. Mendelian Randomization and SMR Analyses

Two-sample MR used TwoSampleMR v0.5.6 [56] and followed established MR methodological guidelines [57,58]. Instrumental variables were selected at *p* < 5 × 10^−8^, r^2^ < 0.001 and kb = 10,000. The inverse-variance weighted (IVW) method served as the primary analysis and was complemented by Cochran’s Q, the MR-Egger intercept and leave-one-out analyses; F statistics >10 were used to assess instrument strength, and reverse MR was performed in parallel. SMR v1.3.1 tested associations between gene expression and disease using brain cis-eQTL meta-analysis data (BrainMeta v2 [59]) as instruments [60], with the heterogeneity in dependent instruments (HEIDI) test used to distinguish pleiotropy from linkage.

### 4.5. Diagnostic Model Construction and Validation

LASSO (glmnet) [61] and SVM-RFE [62] were intersected to define the signature, an L1-regularized logistic regression model was fitted, and AUCs were computed with pROC [63]. Internal validation used leave-one-out cross-validation (LOOCV): in each fold the 33 within-fold samples were standardized and used for modeling, the held-out sample was predicted, and held-out predictions from all 34 samples were pooled. External validation applied the training-set threshold throughout, with reporting guided by the TRIPOD statement [64].

### 4.6. Drug Repurposing and Molecular Docking

Drawing on the known transcriptional effects of *TLR4* knockdown and simvastatin treatment in the Library of Integrated Network-Based Cellular Signatures (LINCS) L1000 dataset [65], we constructed *TLR4*/NF-κB pathway response gene panels for the two perturbations (14 genes each; Supplementary Table S6) and tested their enrichment among genes upregulated in the TLE hippocampus by GSEA, with NES >1 and FDR < 0.05 as significance criteria [66]. Candidate drugs were screened by DSigDB dual-input enrichment analysis [67]. Docking used the TLR4/MD-2 crystal structure (Protein Data Bank [PDB]: 3FXI) [68] as the receptor with AutoDock Vina [69]; the pocket was defined at the MD-2 hydrophobic ligand-binding cavity [70]. TAK-242 was docked only as an exploratory reference compound; because it inhibits TLR4 through an intracellular TLR4 binding mechanism rather than the MD-2 pocket [29], it was not treated as a positive control for MD-2 binding.

### 4.7. Statistical Analysis

Analyses were conducted in R v4.4.3. Multiple testing was controlled by FDR throughout: differential expression, Milo and GSEA used FDR < 0.05, and enrichment analyses used adjusted *p* < 0.05. Microbiome MR was FDR-corrected, and nominal *p* values are reported for metabolite MR. Single-cell statistics were computed per individual. MR reporting follows the STROBE-MR statement [71].

## 5. Conclusions

Through integrated transcriptomic and genetic epidemiological analyses, we prioritized candidate neuroimmune hub genes, including *TLR4*, in drug-resistant TLE and delineated a candidate microglia–vascular endothelium–peripheral myeloid inflammatory axis. Plasma kynurenine showed a nominal genetic-epidemiological association with focal epilepsy that requires pathway-level confirmation. Peripheral blood *TLR4* showed exploratory adjunctive diagnostic potential in drug-naïve newly diagnosed patients, and simvastatin emerged as a computationally predicted repurposing candidate. These findings are hypothesis-generating and require prospective cohort validation, direct barrier and cell-state measurements, and functional experiments before clinical or mechanistic conclusions can be drawn.

## Data Availability

All datasets analyzed in this study are publicly available. The bulk RNA-seq datasets are available in the Gene Expression Omnibus (GEO) under accession numbers GSE134697 (paired discovery cohort of drug-resistant TLE hippocampus and temporal neocortex), GSE256068 (external hippocampal validation cohort), and GSE143272 (peripheral blood validation cohort). The integrated single-cell/nucleus RNA-seq atlas was constructed from two public GEO datasets: GSE190452 (nuclear RNA profiles from four drug-resistant TLE patients and four non-epileptic controls; NovaSeq 6000) and GSE140393 (five additional unsorted single-cell RNA-seq samples from drug-resistant TLE surgical tissue; HiSeq 4000). Genome-wide association study summary statistics were obtained from the MiBioGen consortium at https://mibiogen.gcc.rug.nl/ (accessed on 3 November 2026), the International League Against Epilepsy (ILAE) focal epilepsy GWAS, and the IEU OpenGWAS project (blood metabolite ID: met-a-375) at https://gwas.mrcieu.ac.uk/ (accessed on 3 November 2026). Brain cis-eQTL data were obtained from BrainMeta v2. Drug–gene association signatures were obtained from DSigDB and the LINCS L1000 database; gene set annotations were obtained from MSigDB, gutMGene, STRING, and the Human Protein Atlas. The primary analysis code, including a parameterized 17-script pipeline with data download and run instructions, is publicly available on GitHub at https://github.com/dmuliuzhenglin-ai/R/tree/main/TLE-TLR4-pipeline (accessed on 3 November 2026). The code and output tables for the revision-stage network sensitivity analysis are available from the corresponding author upon reasonable request.

## Supplementary Materials

The following supporting information can be downloaded at: https://www.mdpi.com/article/10.3390/ijms27219960/s1.
