# Supplementary material for: A TLR4-Associated Microglia–Endothelial Inflammatory Axis in Drug-Resistant Temporal Lobe Epilepsy: Integrated Transcriptomic and Genetic Evidence
Source: Int J Mol Sci. 2026 Nov 6;27(21):9960. doi: 10.3390/ijms27219960 (PMC13764224; doi:10.3390/ijms27219960)
Supplement: Supplementary file 1 [file ijms-27-09960-s001.zip › Supplementary_figures_S1-S11_EN.pdf]

# Supplementary material

## Supplementary figures

Supplementary Fig. S1: Overview of differential expression analysis (volcano plot, PCA, and heatmap of the top 50 differentially expressed genes).

Supplementary Fig. S2: WGCNA module–trait correlation heatmap.

Supplementary Fig. S3: Dot plot of the expression of the five hub genes and EDIL3 across brain cell types (grouped by control/TLE).

Supplementary Fig. S4: Dot plot of marker expression in microglia after dual filtering.

Supplementary Fig. S5: Dot plot of lineage marker expression across the 16 cell types of the snRNA-seq atlas (annotation quality check).

Supplementary Fig. S6: Exploratory microbiome MR scan results (screening volcano plot of 203 taxa and forest plot of 9 nominally significant taxa; none statistically significant after BH correction).

Supplementary Fig. S7: Supplementary results of microglia-to-endothelium communication (bubble plots of the top 15 ligand–receptor pairs and bar plots of communication probability in each group).

Supplementary Fig. S8: ROC curve of LOOCV internal validation of the TGFB1+TLR4 two-gene diagnostic signature in the training cohort.

Supplementary Fig. S9: Venn diagram of differential gene overlap between the discovery cohort and the independent GSE256068 cohort (TLE-HS vs normal hippocampus).

Supplementary Fig. S10: Bar plots of DSigDB dual-input drug enrichment (simvastatin significantly enriched in both the five-hub-gene input and the upregulated-DEG input).

Supplementary Fig. S11: Dot plot of the expression of simvastatin-related targets (TLR4, LY96, TNF, NFKB1, CCL2, HMGCR) across the 16 cell types, grouped by control/TLE.

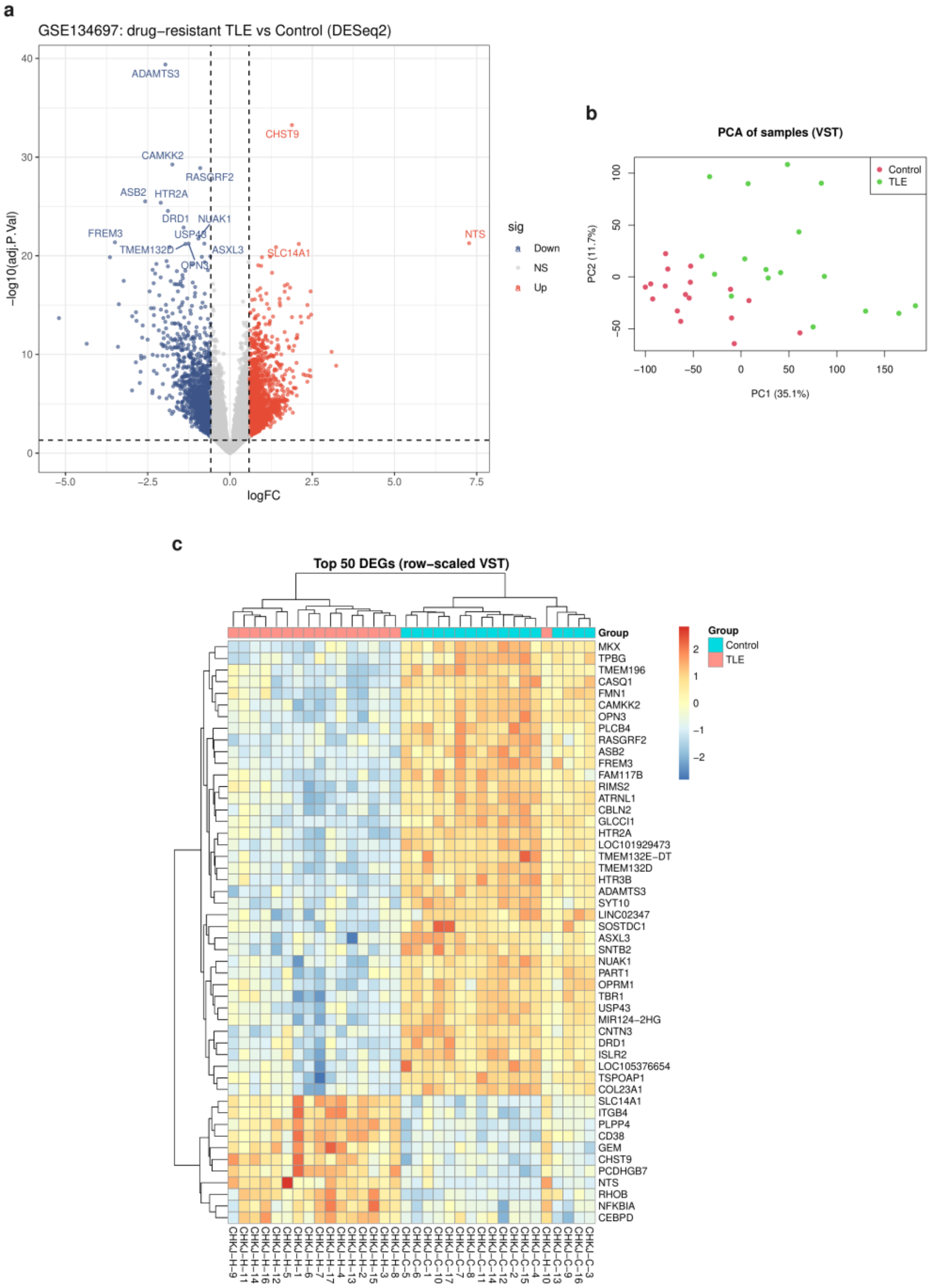


*Supplementary Figure S1. Overview of differential expression analysis (volcano plot, PCA, and heatmap of the top 50 differentially expressed genes).*

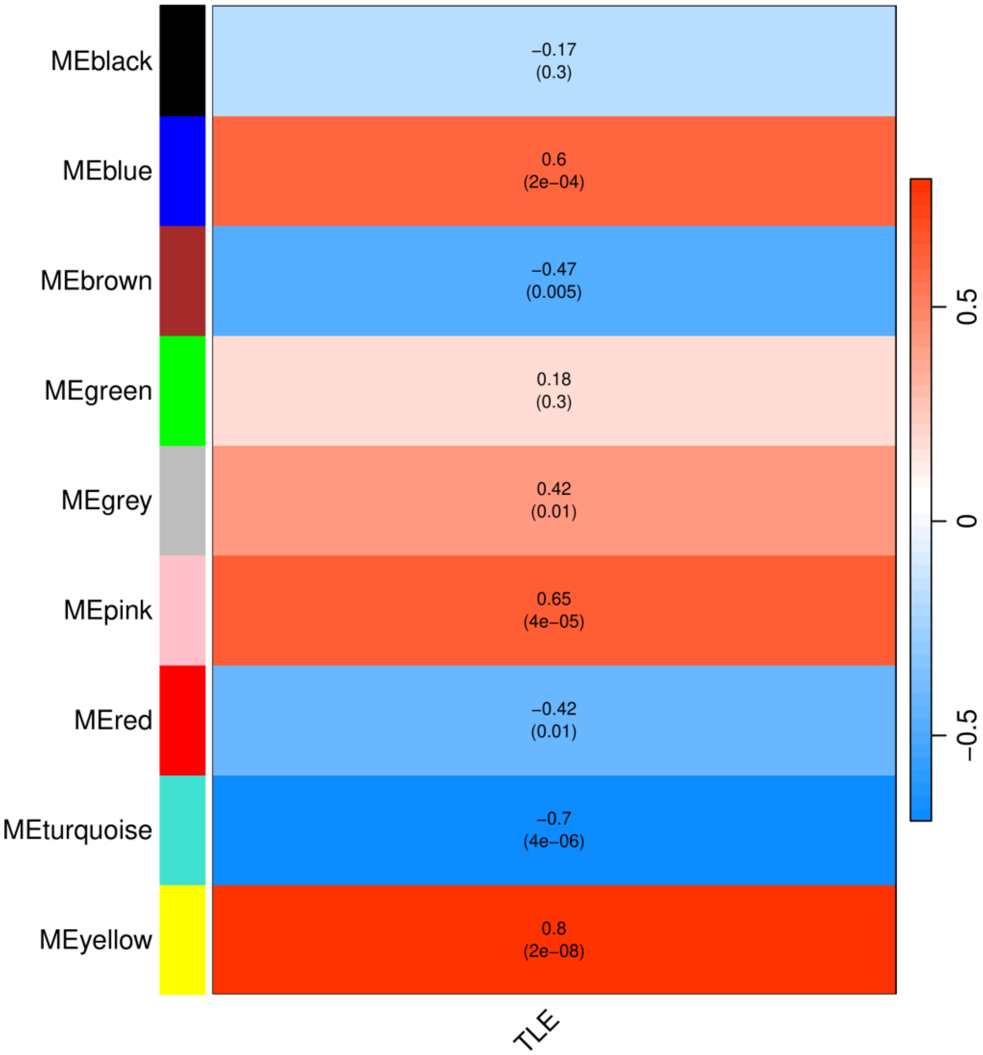


*Supplementary Figure S2. WGCNA module–trait correlation heatmap.*

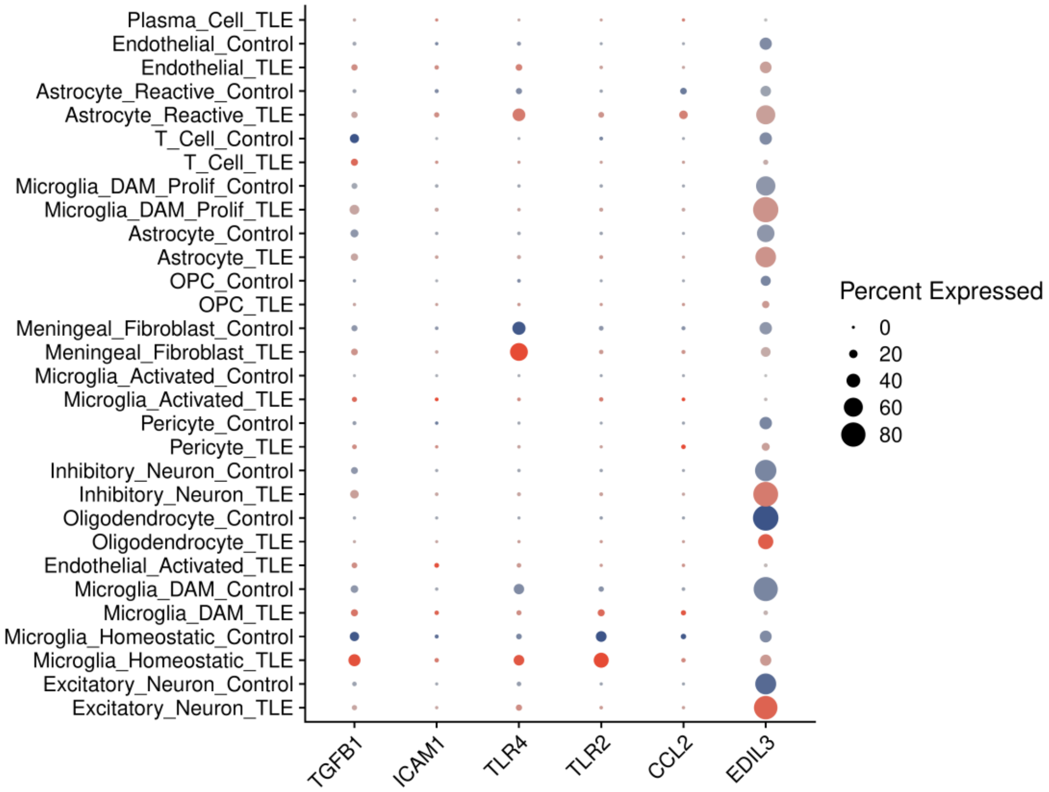


*Supplementary Figure S3. Dot plot of the expression of the five hub genes and EDIL3 across brain cell types (grouped by control/TLE).*

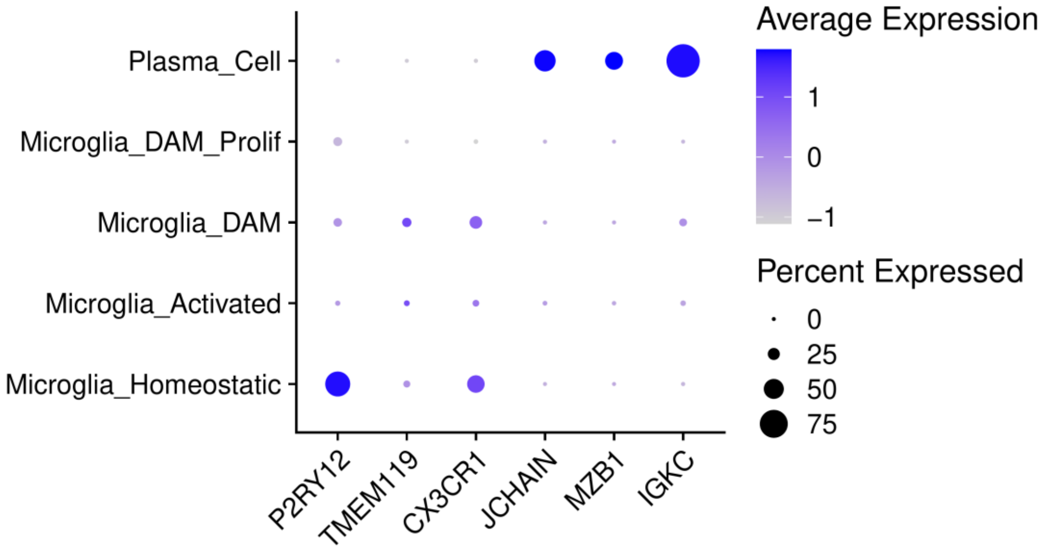


*Supplementary Figure S4. Dot plot of marker expression in microglia after dual filtering.*

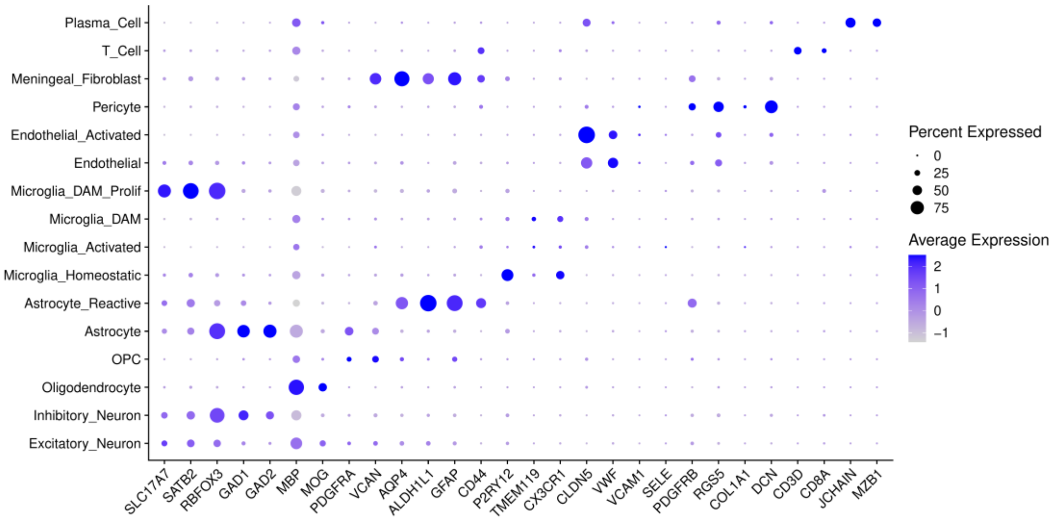


*Supplementary Figure S5. Dot plot of lineage marker expression across the 16 cell types of the snRNA-seq atlas (annotation quality check).*

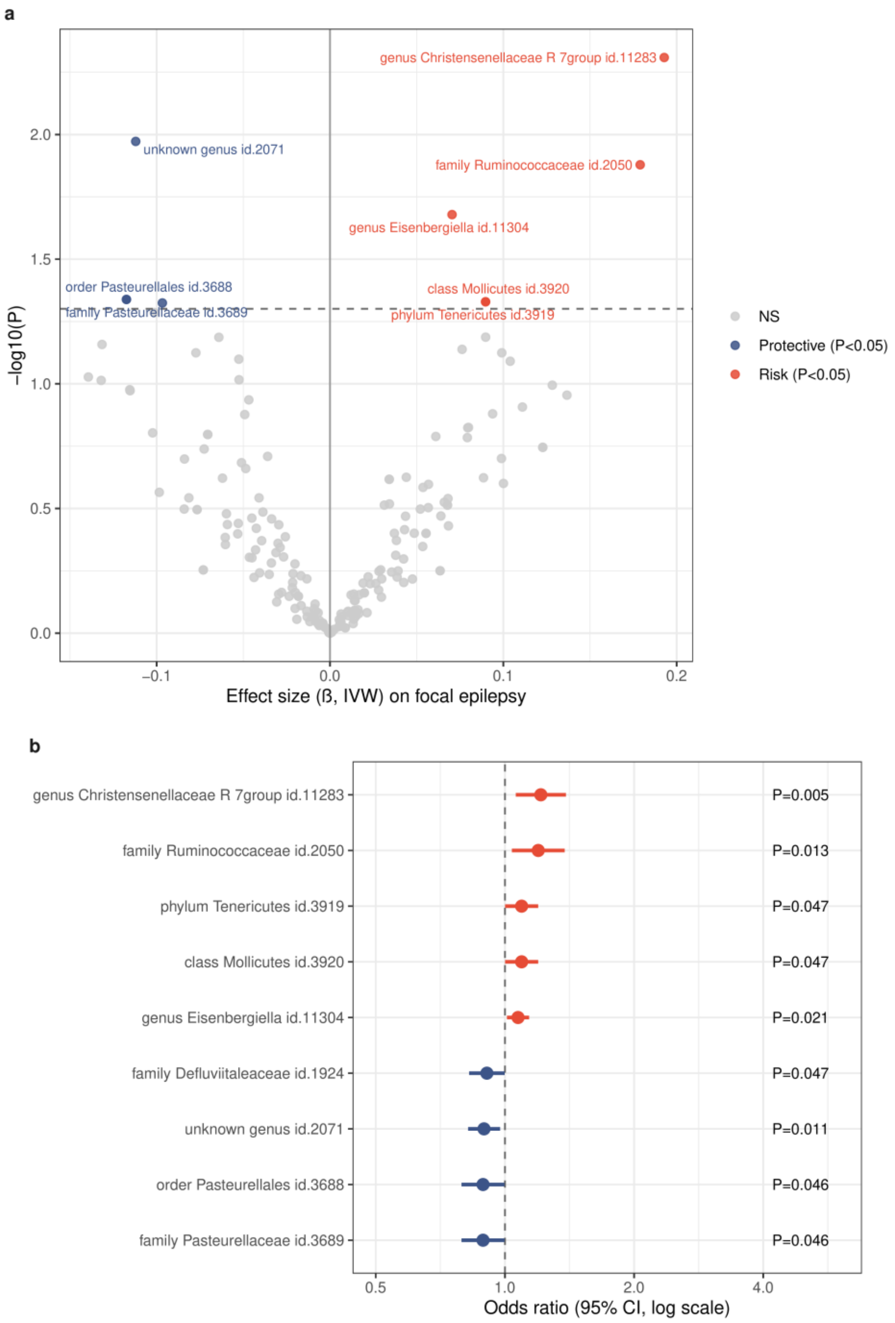


*Supplementary Figure S6. Exploratory microbiome MR scan results (screening volcano plot of 203 taxa and forest plot of nine nominally significant taxa; none statistically significant after BH correction).*

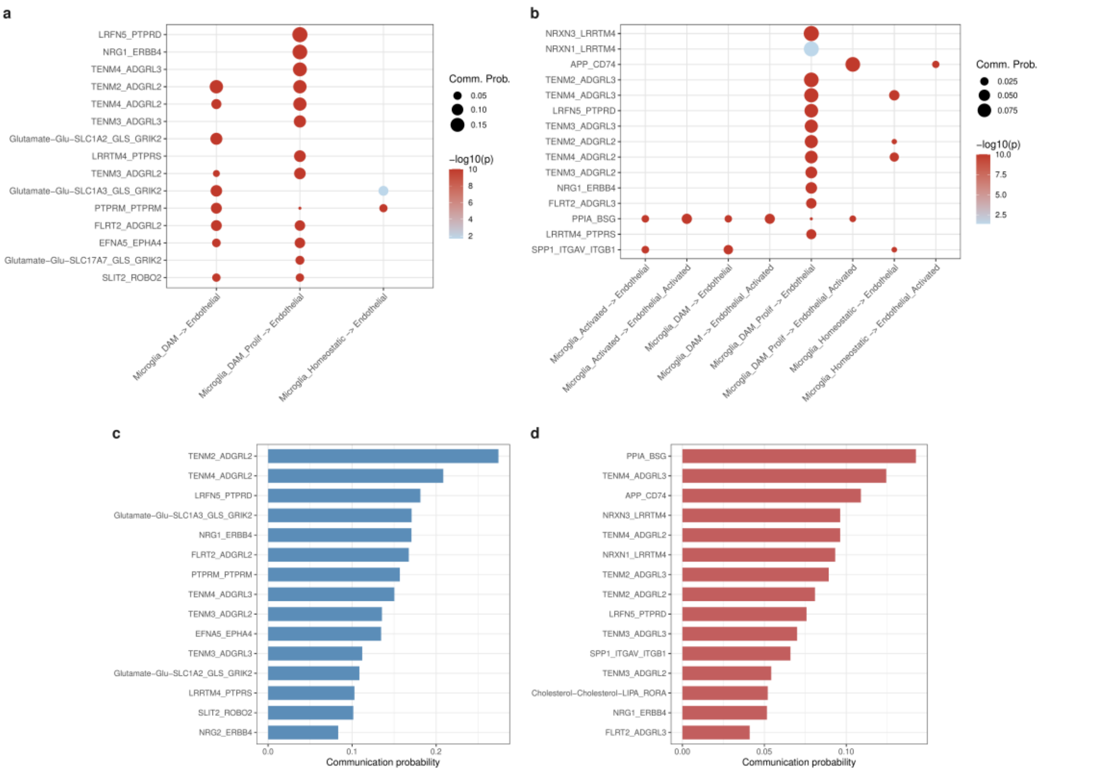


*Supplementary Figure S7. Supplementary results of microglia-to-endothelium communication (bubble plots of the top 15 ligand–receptor pairs and bar plots of communication probability in each group).*

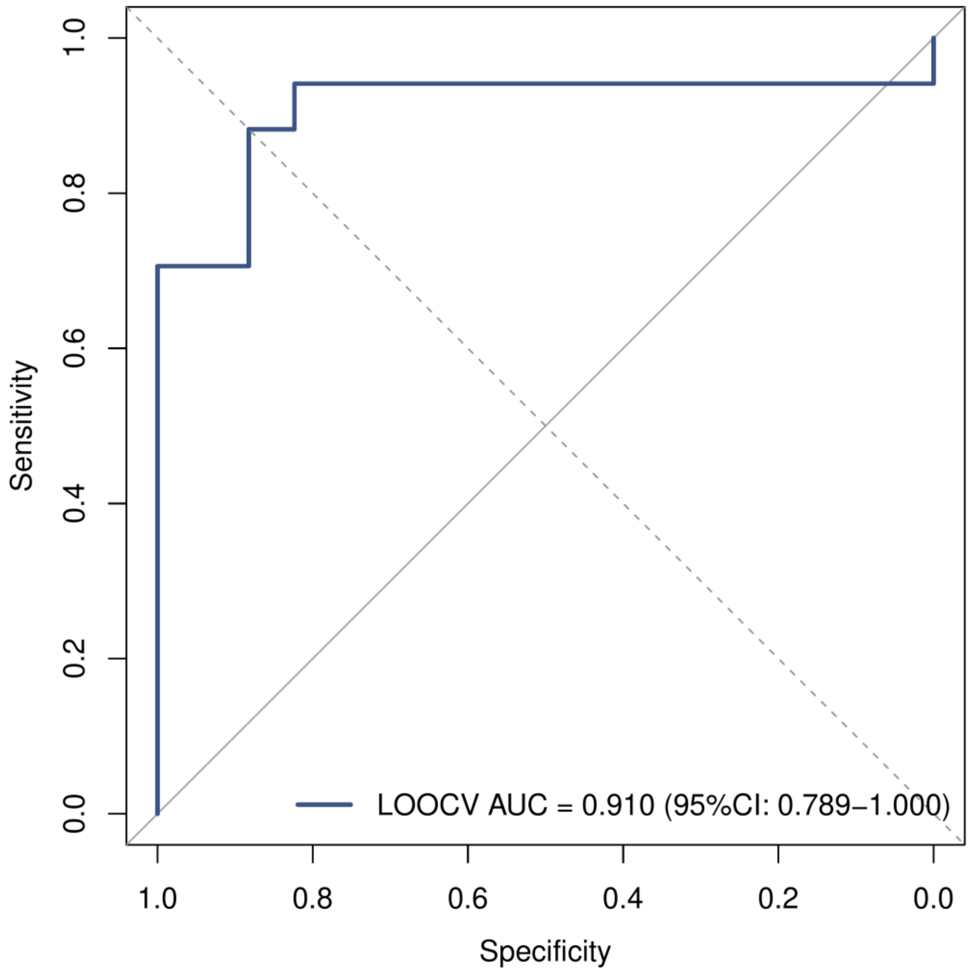


*Supplementary Figure S8. ROC curve of LOOCV internal validation of the TGFB1+TLR4 two-gene diagnostic signature in the training cohort.*

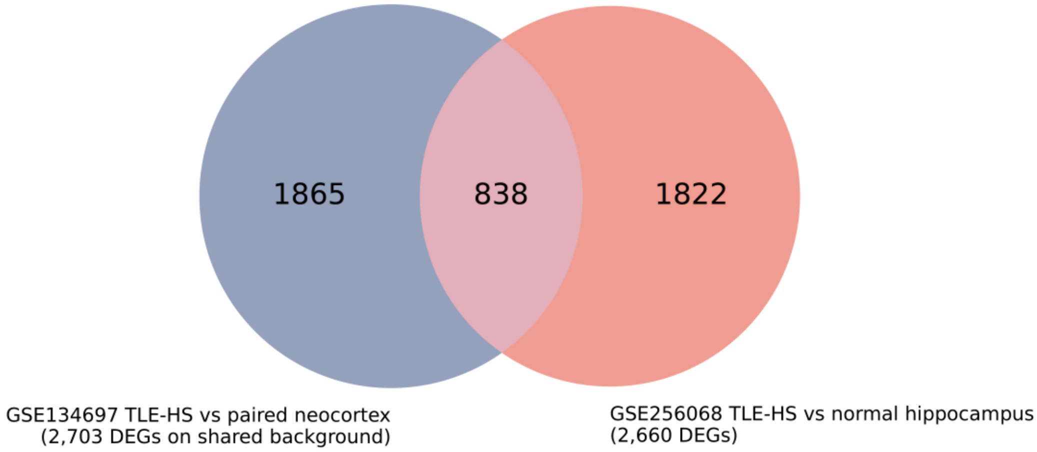


*Supplementary Figure S9. Venn diagram of differential gene overlap between the discovery cohort and the independent GSE256068 cohort (TLE-HS vs normal hippocampus).*

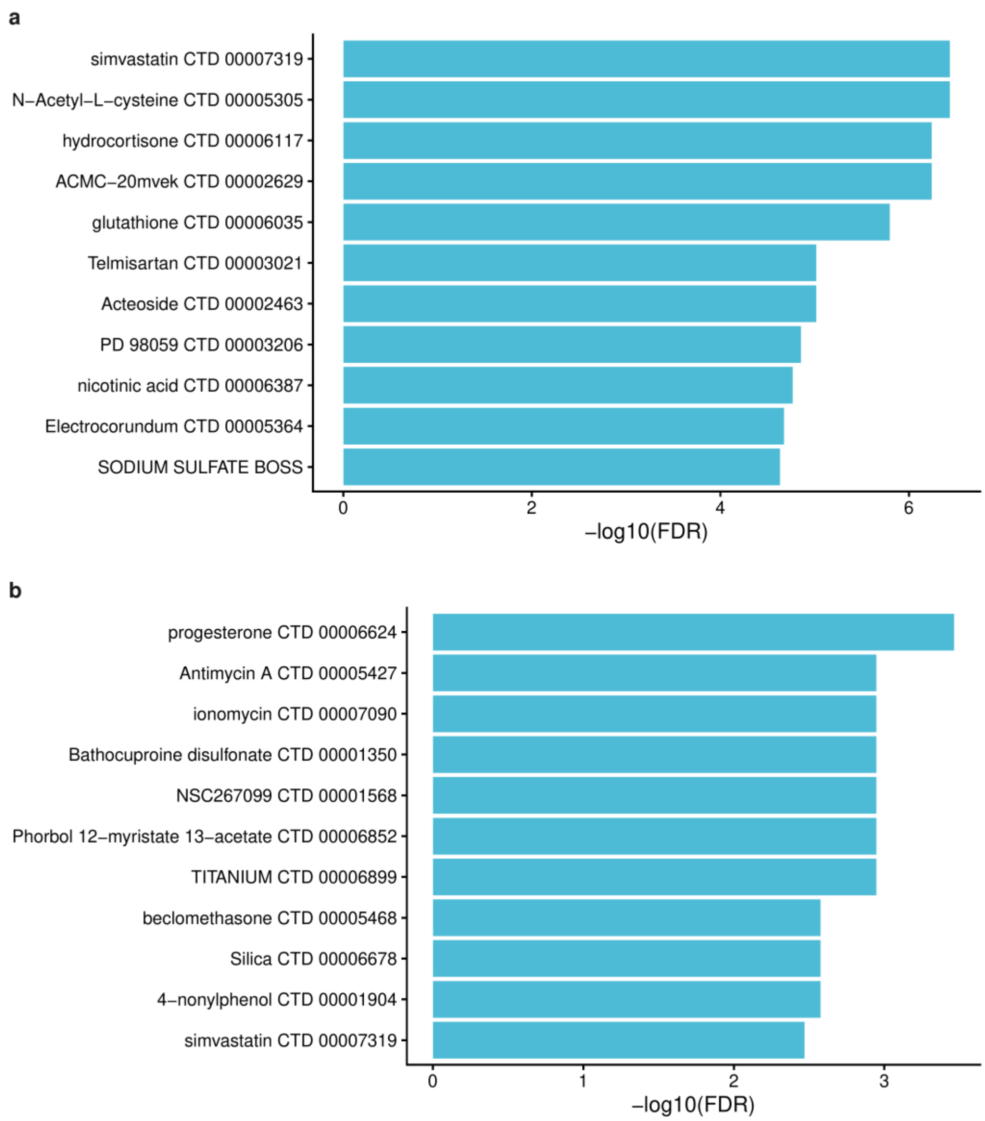


*Supplementary Figure S10. Bar plots of DSigDB dual-input drug enrichment (simvastatin significantly enriched in both the five-hub-gene input and the upregulated-DEG input).*

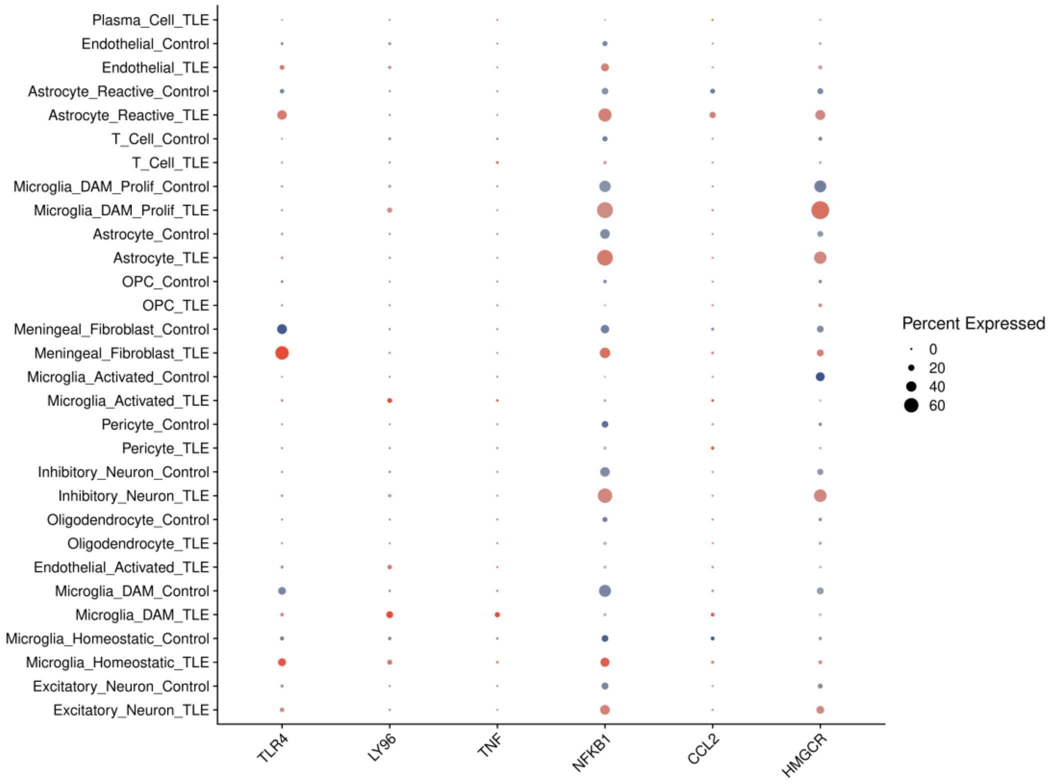


*Supplementary Figure S11. Dot plot of the expression of simvastatin-related targets (TLR4, LY96, TNF, NFKB1, CCL2, HMGCR) across the 16 cell types, grouped by control/TLE.*
